# The Effect of Chitosan Type on Biological and Physicochemical Properties of Films with Propolis Extract

**DOI:** 10.3390/polym13223888

**Published:** 2021-11-10

**Authors:** Karolina Stanicka, Renata Dobrucka, Magdalena Woźniak, Anna Sip, Jerzy Majka, Wojciech Kozak, Izabela Ratajczak

**Affiliations:** 1 Department of Chemistry, Faculty of Forestry and Wood Technology, Poznan University of Life Sciences, Wojska Polskiego 75, 60625 Poznań, Poland; karolina.stanicka@up.poznan.pl (K.S.); magdalena.wozniak@up.poznan.pl (M.W.); 2 Department of Industrial Products and Packaging Quality, Institute of Quality Science, Poznań University of Economics and Business, al. Niepodległości 10, 61875 Poznań, Poland; renata.dobrucka@ue.poznan.pl (R.D.); wojciech.kozak@ue.poznan.pl (W.K.); 3 Department of Biotechnology and Food Microbiology, Faculty of Food Science and Nutrition, Poznan University of Life Sciences, Wojska Polskiego 48, 60627 Poznań, Poland; anna.sip@up.poznan.pl; 4 Department of Wood Science and Thermal Technics, Faculty of Forestry and Wood Technology, Poznan University of Life Sciences, Wojska Polskiego 38/42, 60627 Poznań, Poland; jerzy.majka@up.poznan.pl

**Keywords:** chitosan, propolis, edible films, antimicrobial activity, sorption, DVS, GAB model

## Abstract

The aim of the research was to determine the influence of chitosan type and propolis extract concentration on biological and physicochemical properties of chitosan-propolis films in terms of their applicability in food packaging. The films were prepared using three types of chitosan: from crab shells, medium and high molecular weight and propolis concentration in the range of 0.75–5.0%. The prepared polysaccharide films were tested for antimicrobial properties, oxygen transmission rate (OTR) and water vapor transmission rate (WVTR). Moreover, sorption tests and structural analysis were carried out. Microbiological tests indicated the best antimicrobial activity for the film consisting of high molecular weight chitosan and 5.0% propolis extract. Both the type of chitosan and propolis concentration affected transmission parameters—OTR and WVTR. The best barrier properties were recorded for the film composed of high molecular weight chitosan and 5.0% propolis extract. The results of sorption experiments showed a slight influence of chitosan type and a significant effect of propolis extract concentration on equilibrium moisture content of tested films. Moreover, propolis extract concentration affected monolayer water capacity (Mm) estimated using the Guggenheim, Anderson and de Boer (GAB) sorption model. The obtained results indicate that chitosan films with an addition of propolis extract are promising materials for food packaging applications, including food containing probiotic microorganisms.

## 1. Introduction

The global production of plastics has been growing dramatically for years. Its rapid growth has been caused by such factors as easy access to the raw material and improved living standards leading to increased consumption. Within the next few years, the packaging industry, in particular the plastic packaging segment, will have to face challenges resulting from the regulations set out in Directive 2019/904 of the European Parliament and the Council [1] on the reduction of the impact of certain plastic products on the environment. The initiative presented in the Directive is also regarded in the broader context of the European Union’s transition to circular economy, which is one of key development areas for the European Union. It is the consequence of the observed environmental degradation, the development of climate policies and the resulting new environmentally-friendly regulations, as well as the increase in awareness and consumers’ expectations of the manner of conducting business. Circular economy related to the biological and technical cycle is governed by three main rules: preservation and enrichment of natural capital by controlling limited stocks and balancing the streams of renewable resources; optimization of the use of raw materials by maintaining products, components and materials in circulation, while keeping their highest usability in both cycles; and development of system efficiency by identifying and removing negative external effects. To meet the expectations resulting from the Directive, circular economy and zero waste approach, manufacturers of plastic packaging are facing enormous changes, and even a kind of revolution. The answer to the search for new polymeric materials are natural materials.

Food packaging is primarily intended to protect the packed food, preserve it, and enable transport. Edible packaging allows extending of the shelf life of food and maintaining its high quality [2]. There are three types of edible packaging. The first is the edible coating, which is mostly used for semi-wet food and for fruit coating. It can also distinguish edible films, which are a thin and continuous layer. It arises as a result of the interaction of polymer chains. Encapsulation is a third type of edible packaging [3]. One of the biggest challenges in the production of edible packaging is the creation of materials with the same properties as those made of plastics. The most commonly used raw materials for the production of edible packaging are starch, gelatin and chitosan [4,5,6].

Chitosan as a natural polymer, is studied for its possible application as packing materials, mainly because of its similarity to biomolecules as well as its biocompatibility, immunological properties, biodegradability and other characteristics [7]. It is produced by means of deacetylation of chitin sourced commercially from shrimp and crab shells [8]. The literature has reported that chitosan in the form of a solution exhibits microbial effects against various strains of microorganisms [8,9]. The proposal to apply chitosan in food packaging is mainly based on its antimicrobial properties exhibited against pathogenic and spoilage microbiota [10,11,12,13]. However, some works, apart from chitosan, use many active ingredients that are incorporated directly into the polymer matrix, such as flavors and antimicrobial and antioxidant compounds or substances, which contribute to product integrity preservation and extension of shelf life. It can be seen as the use of natural additives, such as plant and animal proteins, polysaccharides, antimicrobial peptides, such as nisin and divergicin, as wells as oils and plant extracts [11,14,15,16,17,18]. Examples of improving functionality by enriching film-forming materials are the use of inorganic nanoparticles of iron or silver oxide and organic nanoforms of cellulose and chitosan. Composites enriched in this way show better optical, mechanical and barrier properties and also biological activity [2,6]. The literature data have shown that ginkgo leaf extract has strong antioxidant activity in gelatin membranes. An important role is also played by anthocyanins, which in packaging materials act as a reducing agent and oxygen suppressor, and essential oils (mainly oregano oil), which are rich in phenolic, terpene and terpenoid compounds. Inorganic nanoparticles can be used to eliminate characteristic odors that result from the use of essential oils. An example is the use of ZnO nanoparticles to reduce the odor of carvacrol. The literature also provides information on the possibility of producing active and intelligent packaging from natural polymers by incorporating sensor materials into the film matrix, such as, e.g., pH indicators developed with the use of anthocyanins or gas levels. Which in the future will allow for providing information about the condition of packaged food [2].The world literature also describes some examples of films based on chitosan and propolis [19,20,21,22]. Propolis is a natural resinous material produced by honeybees from various plant sources [23,24]. The antibacterial and antifungal activity is the main function of its extracts, but propolis also shows anticancer, antioxidant and hepatoprotective properties [25,26,27,28,29,30]. Propolis is characterized by a diverse and complex chemical structure, but the biological activity of its extracts is mainly attributed to phenolic compounds [23,24,30,31]. The components of propolis with antimicrobial activity include such phenolic compounds as pinostrobin, pinocembrin, caffeic acid phenethyl ester (CAPE), galangin, apigenin and coumaric acid [27,30,32,33,34]. Propolis obtained from Poland is also a rich source of phenols, as it contains, among others, apigenin, galangin, naringenin, chrysin, pinocembrin, kaempferol, caffeic acid, coumaric acid and cinnamic acid [35,36,37,38]. As an antibiotic, antifungal and antibacterial agent, propolis has been added to formulations used in films or coatings in order to minimize spoilage and extend the shelf life of fruit and vegetables [22,39]. It was proven, e.g., by studies conducted by Alvarez et al. [40], who tested antimicrobial properties of chitosan films with propolis added as a bioactive compound. In vitro assays showed an inhibitory effect on *Escherichia coli* and *Listeria monocytogenes*. A bacteriostatic-effect chitosan and propolis extract could be observed from in vivo application of coatings on freshly cut broccoli [40]. Furthermore, Siripatrawan and Vitchayakitti [21] studied chitosan films containing different concentrations of propolis extract. They observed that the studied films exhibited antimicrobial effects, while the mechanical and barrier properties of the films were improved by the addition of propolis extract. In turn, Correa-Pacheco et al. [22] showed that the application of chitosan nanoparticles and ethanolic extract of propolis in edible films is a promising alternative for active packaging with antibacterial properties, given their use for the preservation of strawberries against foodborne bacteria.

In this study chitosan-propolis films were prepared using different concentrations of propolis extract and three types of chitosan: from crab shells, medium and high molecular weight. The aim of the work was to determine the influence of chitosan type and propolis extract concentration on biological and physicochemical properties of the obtained films. To the best of our knowledge, the relationship between the type of chitosan and different concentrations of Polish propolis extract and their influence on the biological and physicochemical parameters of the obtained films has not yet been investigated. The biological activity of chitosan-propolis films was determined by agar diffusion method against strains of pathogenic or potentially pathogenic bacteria and yeasts. The barrier properties of tested films were assessed by analysis of oxygen transmission rate (OTR) and water vapor transmission rate (WVTR). In turn, the sorption parameters of films were determined using a dynamic vapor sorption (DVS) apparatus and based on these results the adsorption isotherms were calculated by the Guggenheim, Anderson and de Boer (GAB) sorption model. Moreover, attenuated total reflectance Fourier transform infrared spectroscopy was used to determine the changes in the structure of films.

## 2. Materials and Methods

### 2.1. Chitosan Films Preparation

Three types of chitosan purchased from Sigma Aldrich (Darmstadt, Germany) were used to prepare the films: from crab shells highly viscous, medium molecular weight (75–85% deacetylated, molecular weight: 90,000–310,000 Da) and high molecular weight (>75% deacetylated, molecular weight: 310,000–375,000 Da). The chitosan film-forming solutions were prepared by dissolving 4 g of chitosan in 400 mL of a 3% acetic acid solution (Avantor Performance Materials, Gliwice, Poland). In addition, 4 mL of glycerin (Chempur, Piekary Śląskie, Poland) as plasticizer were added to each solution. The final solutions were homogenized using a homogenizer and poured into Petri dishes with Teflon liners. The films were then dried at room temperature.

### 2.2. Chitosan-Propolis Films Preparation

The chitosan film-forming solutions obtained as described in Section 2.1 were used to prepare chitosan-propolis films. Each chitosan solution (100 mL) was mixed with 1.5 mL of Tween-20 (Sigma Aldrich, Darmstadt, Germany) and the ethanolic extract of propolis (EEP) (PROP-MAD, Poznań, Poland) to obtain final propolis concentrations of 0.75%, 2.5% and 5.0%, respectively. The final solutions after homogenization were poured into Petri dishes with Teflon liners and the films were dried at room temperature. As a result of the experiment, 12 different film samples were obtained, the denotations of which are presented in Table 1.

### 2.3. Antimicrobial Properties of Films

The biological activities of chitosan-based films in the form of discs with a diameter of 1 cm were determined by the agar diffusion method against strains of pathogenic or potentially pathogenic bacteria (*Listeria monocytogenes*, *Staphylococcus aureus*, *Escherichia coli*, *Salmonella* Typhimurium, *Bacillus cereus*), probiotic bacteria (*Lactobacillus paracasei*, *Enterococcus faecium*) and yeasts (*Saccharomyces cerevisiae*). All indicator strains were isolates obtained from the collection of the Department of Food Biotechnology and Microbiology, the Poznan University of Life Sciences. Petri dishes with Mueller Hilton agar medium (GRASO Biotech, Starogard Gdański, Poland) were inoculated with 10^6^ cfu/mL of indicator strains. Then chitosan-based films were placed on the surface of inoculated plates and incubated at 37 °C for 18 h. After incubation, the inhibition zones were evaluated by a computer scanning system (MultiScanBase v14.02).

### 2.4. Oxygen Transmission Rate

The oxygen transmission rate (OTR) analyses were carried out for chitosan and chitosan-propolis films. Oxygen transmission rate was determined in accordance with ASTM F3136-15 [41] by means of the OxyPerm system consisting of an Oxysense 325 analyzer and a dedicated permeation chamber with an in-built OxyDot oxygen sensor (OxySense, Dallas, TX, USA). The system records changes in oxygen concentration in the chamber based on the measurement of fluorescence quenching time in accordance with ASTM F2714-08 [42]. Samples used in the analyses were cut into squares with 6.5 cm sides. During the tests the temperature was 23 °C and relative humidity (RH) was 65%. The measurements were ended when the coefficient of determination between the respective partial measurements exceeded 0.95. All measurements were done in triplicate.

### 2.5. Water Vapor Transmission Rate

The water vapor transmission rate (WVTR) of chitosan-based films was determined on the basis of ISO 2528:2017 [43]. Measuring vessels containing 10 g of anhydrous calcium chloride (Avantor Performance Materials, Gliwice, Poland) were covered with a film sample, tightly sealed and placed in a desiccator containing a saturated sodium chloride solution (Avantor Performance Materials, Gliwice, Poland) (RH = 65%, T = 23 °C). The vessels were then weighed to an accuracy of 0.001 g. The calculations of water vapor transmission rate (WVTR) were based on the following equation:

(1) $$\mathrm{WVTR}\;=\;\frac{m\cdot 24}{A}$$

where: WVTR is the water vapor transmission rate (g/m^2^ × 24 h), *m* (g) is the mass increase and *A* (m^2^) is the water vapor transmission area.

### 2.6. Sorption Experiments

The sorption experiments were performed using a dynamic vapor sorption (DVS) apparatus (DVS Advantage 2, Surface Measurement Systems, London, UK). The equilibrium moisture content (EMC) values were recorded for 7 levels of relative humidity (RH) in the adsorption mode at 25 °C. The set RH values ranged from 0 to 0.80 (i.e., 0, 0.05, 0.20, 0.35, 0.50, 0.65 and 0.80). Prior to the experiments, the prepared chitosan and chitosan-propolis film samples were stored in a desiccator over phosphorus pentoxide (Avantor Performance Materials, Gliwice, Poland) for two weeks leading to an moisture content (MC) close to dry mass. The average dry mass of each sample was ca. 12 mg. Prior to each sorption experiment the pre-dried samples were additionally equilibrated in the DVS apparatus in dry nitrogen. Afterwards, the RH was increased stepwise. It was assumed that the hygroscopic equilibrium was reached at a given air RH value when the mass change was less than 0.002% min^−1^ with the time window of 10 min and the minimum stability period of over 60 min. For each RH step the EMC values were calculated.

### 2.7. Sorption Modelling

The adsorption isotherms were calculated by the Guggenheim, Anderson and de Boer (GAB) equation [44,45]:

(2) $$EMC=M_{m}\frac{K\cdot C\cdot RH}{\left(1-K\cdot RH\right)\cdot \left(1-K\cdot RH+C\cdot K\cdot RH\right)}$$

where: *M_m_* (kg/kg) is the monolayer moisture content, *C* (-) is the equilibrium constant related to the monolayer sorption, *K* (-) is the equilibrium constant related to the multilayer sorption and *RH* (-) is the relative humidity. It is assumed that Equation (2) describes water molecule bonding to sorption sites (monomolecular layer). Water molecules in the monolayer become secondary sorption sites and additional layers of water are formed (multilayer sorption). Coefficients of Equation (2) were estimated using the Levenberg–Marquardt iterative algorithm with SigmaPlot 9.0 software.

### 2.8. Attenuated Total Reflectance Fourier Transform Infrared Spectroscopy

Attenuated total reflectance Fourier transform infrared spectroscopy (ATR-FTIR) was carried out to determine structural interactions of different chitosan types with propolis components. The ATR-FTIR spectra of films were recorded by a Nicolet iS5 spectrophotometer (Thermo Fisher Scientific, Waltham, MA, USA) with Fourier transform and deuterium triglycine sulfate detector. The spectra were recorded at 4000–600 cm^−1^ at a resolution of 4 cm^−1^ recording 32 scans.

## 3. Results and Discussion

### 3.1. Antimicrobial Activity

The antimicrobial activity of chitosan-propolis films against bacteria and yeast was compared with films from chitosan without EEP addition and the results are presented in Table 2.

The results indicated that all examined chitosan films regardless of the chitosan type show no activity against the tested microbial strains. Moreover, none of the chitosan-propolis films restricted growth of Gram-negative bacteria (*E. coli* and *S.* Typhimurium), probiotic bacteria (*Lb. paracasei* and *E. faecium*) and yeast (*S. cerevisiae*). In turn, all variants of chitosan-propolis films showed moderate activity against Gram-positive bacteria (*B. cereus*). The films containing high molecular chitosan and propolis extract at various concentrations (from 0.75 to 5.0%) exhibited activity against two Gram-positive strains—*L. monocytogenes* and *S. aureus.* Moreover, *L. monocytogenes* was the most sensitive bacterial strain against high molecular chitosan films with the propolis extract—the inhibition zone ranged from 17 (H 0.75) to 26 mm (H 2.5 and H 5.0). Moderate activity against *S. aureus* was also observed for films consisting of chitosan from crab shells and medium molecular weight with an addition of the propolis extract at the concentration of 5.0%.

Lack of antimicrobial activity of various chitosan types in the form of films is in agreement with the results presented by Siripatrawan and Vitchayakitti [21], where chitosan films showed no activity against *S. aureus*, *E. coli* and *Pseudomonas aeruginosa*. The literature data indicated that chitosan solutions demonstrated strong antibacterial activity, while films prepared from the same chitosan solutions did not show this beneficial property [9,17,19]. Strong antimicrobial action of chitosan solution is connected with its acidity and the positive charge on the C-2 position of glucosamine monomer at pH below 6.0, which would support the interaction of chitosan chains with anionic groups of bacterial cell surfaces [9,12,46]. In turn, the lack of bactericidal activity of chitosan films would be connected with the fact that this form of chitosan is incapable of diffusing through the agar media and interact with microbial cell walls, thus failing to exhibit bactericidal properties [19].

The addition of propolis extract to chitosan caused an enhanced antibacterial activity of films, which is in agreement with literature data [19,21,47]. The antimicrobial activity of propolis extracts collected from various geographical regions was confirmed in many scientific reports [32,33,34,48,49]. According to literature, propolis extracts showed activity against Gram-positive bacteria used in this study (*L. monocytogenes*, *S. aureus* and *B. cereus*) and also Gram-negative bacteria (*E. coli* and *S.* Typhimurium). However, Gram-negative bacteria exhibited lower sensitivities to propolis than Gram-positive bacteria [30,32,48,49,50,51,52]. In addition, the chemical composition and biological activity of propolis extracts depend on various factors, including geographical origin, time and method of harvesting, as well as the solvent used in the extraction of the raw material [31,38,53,54]. In turn, the impact of propolis extract on probiotic bacteria is dependent on the bacteria strain and propolis concentration [55,56]. The lack of activity of chitosan-propolis films against probiotic bacteria could be used to produce packaging for foodstuffs containing probiotic microorganisms. Furthermore, the antifungal activity of propolis extract has been described in the literature, where the effect of propolis on various strains of fungi has been confirmed [35,38,48,52]. The lack of chitosan-propolis films activity against *S. cerevisiae* observed in this study could be connected with low propolis concentrations, because according to literature data, fungi are characterized by greater resistance to propolis compared to bacteria [35,49,52].

The results described by Siripatrawan and Vitchayakitti [21] indicated that chitosan-propolis films showed lower activity against *S. aureus* than paper discs containing a propolis extract. According to the authors, a possible reason for the reduction in the effectiveness of propolis incorporated into chitosan chains compared to pure propolis extract may result from the close interaction between chitosan functional groups (amine and hydroxyl) with phenolic compounds that are responsible for the biological activity of propolis. Therefore, phenols are not able to diffuse through the agar medium and thus inhibit the growth of bacteria [21]. The results presented in Table 2 indicated that films consisting of propolis extract and high molecular weight chitosan showed greater activity against Gram-positive bacteria strains compared to other chitosan-propolis films. It could be connected with the fact that high molecular weight chitosan has a compact structure due to strong intramolecular hydrogen bonds [57], therefore phenolic compounds from propolis extract may be less bound to the chitosan matrix and thus may diffuse through the agar medium and inhibit bacterial growth.

### 3.2. Oxygen Transmission Rate

**Figure 1:**
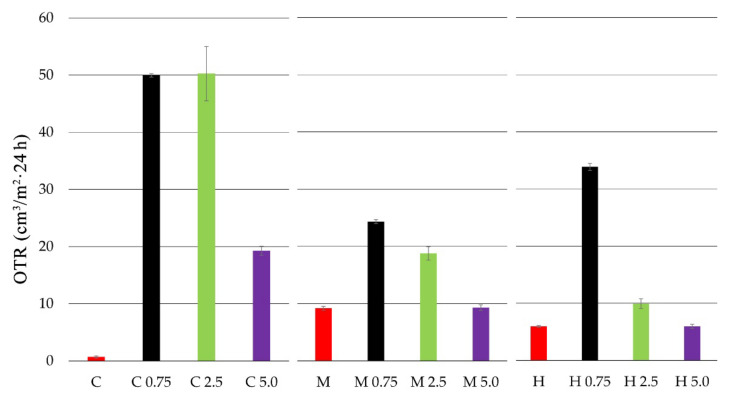
Oxygen transmission rate (OTR) of chitosan films with propolis extracts at various concentrations.

The oxygen transmission rate (OTR) for chitosan and chitosan-propolis film samples is presented in Figure 1.

Based on the conducted studies, it was observed that the film from pure crab shell chitosan (C) was characterized by the lowest oxygen transmission rate. The highest OTR values, and thus the lowest barrier properties, were exhibited by the medium molecular weight chitosan film (M). The addition of propolis extract at each concentration caused an increase in the OTR value for all tested chitosan films. In the case of crab shell chitosan film, the addition of propolis extract at 0.75% and 2.5% caused a significant increase in OTR for up to about 50 (cm^3^/m^2^ × 24 h). A similar correlation (OTR increase) was observed for the medium molecular weight chitosan film. In this case, propolis extract addition at the concentrations of 0.75% and 2.5% caused the OTR value to increase to 24 and 18 (cm^3^/m^2^ × 24 h), respectively. Generally, the high molecular weight chitosan film exhibited low OTR values. However, with the addition of 0.75% propolis extract, the OTR value increased to 33 (cm^3^/m^2^ × 24 h).

### 3.3. Water Vapor Transmission Rate

**Figure 2:**
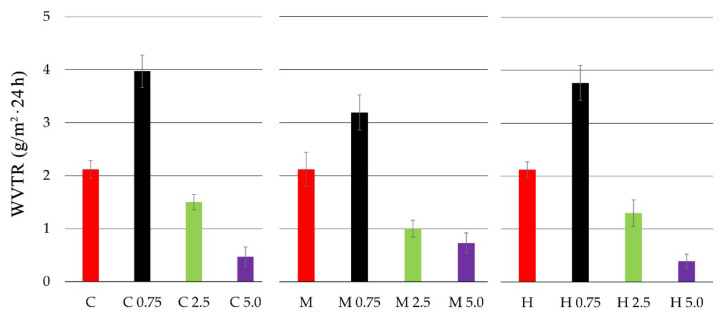
Water vapor transmission rate (WVTR) of chitosan films with propolis extracts at various concentrations.

The water vapor transmission rate (WVTR) for chitosan and chitosan-propolis film samples is presented in Figure 2.

All tested pure chitosan films (C, M, H) presented similar values of water vapor transmission rate (WVTR). The highest WVTR values were observed for the films of all chitosan types with propolis extract at 0.75% concentration. The WVTR values for chitosan films with 0.75% EEP was 3.97 (g/m^2^ × 24 h) for C 0.75, 3.19 (g/m^2^ × 24 h) for M 0.75 and 3.76 (g/m^2^ × 24 h) for H 0.75. In turn, the addition of 2.5% and 5.0% propolis extracts caused lower WVTR values. The film containing high molecular weight chitosan with 5.0% EEP was characterized by the lowest WVTR values—0.39 (g/m^2^ × 24 h). The obtained results indicate that the concentrations of propolis affect the value of the WVTR parameter.

As propolis extract is a chemically complex resinous bee product containing flavonoids, phenolic acids, waxes, essential oils and various organic compounds, its addition to chitosan caused an increase in the WVTR and OTR values at the concentrations of 0.75% and 2.5%, i.e., lower barrier properties of films. However, the 5.0% addition propolis extract caused the opposite effect, where the WVTR and OTR values were reduced (better barrier properties), which could be the result of film matrix consolidation through the increase of termolecular impacts and cross-linking between the ingredients. Similarly, Siripatrawan and Vitchayakitti [21] observed that propolis extract reduces permeability of water vapor (about 28%) and oxygen (62%), but increases the tensile strength and elongation at break of chitosan films with the addition of propolis. The enhanced mechanical properties can be related to the strong interaction between chitosan and phenolic ingredients present in propolis, such as quercetin, naringenin and baicalin [58].

### 3.4. Sorption Behavior Derived from Experimental GAB Sorption Isotherms

**Figure 3:**
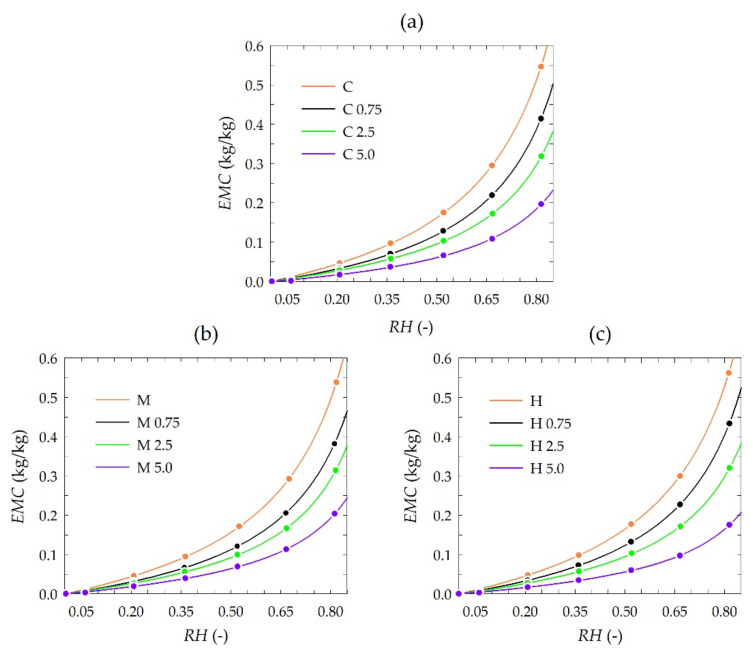
Experimental adsorption data (dots) and results of modeling (lines) with the GAB model (Equation (2)) of chitosan at 25 °C depending on propolis extract concentration: (**a**) crab shells, (**b**) medium molecular weight, (**c**) high molecular weight.

The results of sorption experiments are presented in Figure 3. Each plot consists of sets of isotherms of chitosan depending on the propolis extract concentration. The isotherms were plotted by fitting the GAB model to each set of experimental data, i.e., measured EMC values are also depicted in the plots.

The equilibrium moisture content (EMC) was generally similar to that reported for pure chitosan and chitosan-methylcellulose composite films [59]. The results of sorption experiments showed a slight influence of the chitosan type, i.e., from crab shells, medium and high molecules weight, on EMC, whereas EMC was significantly influenced by the concentration of used propolis extract. A distinct reduction of EMC values was observed for higher concentrations of propolis extract. Observed differences in EMC were detailed by modeling sorption isotherms.

The estimated values of coefficient in the GAB model for all chitosan type variants and used concentrations of propolis extract are presented in Table 3.

Concentration of propolis extract in the range from 0 to 5.0% affected monolayer water capacity (M_m_) estimated using the GAB model. The values of the M_m_ coefficient were reduced to 39.2% (from 0.176 to 0.041 kg/kg) for chitosan from crab shells and 40.9% (from 0.159 to 0.065 kg/kg) and 32.7% (from 0.171 to 0.056 kg/kg) for medium and high molecular weight chitosan, respectively (Table 3). As a result of the increase in propolis extract concentration, the accessibility of primary sorption sites is limited. The described effect applies to all types of chitosan films. This supports the hypothesis that an increase in propolis extract concentration reduces active sorption sites.

The comparison of estimated values of C and K parameters indicate that water molecules are organized in a monolayer with water molecules strongly bound for all examined types of chitosan and to a degree independent of the propolis extract concentration used. Simultaneously in all studied types of chitosan and used propolis extract concentrations water molecules organized in a multilayer do differ considerably from bulk liquid molecules [60].

The estimated values of the C parameter were always slightly higher than K in the corresponding isotherms (Table 3). This suggests that monolayer water molecules are bound much more strongly than those with multilayer bonding [61]. Moreover, slightly higher values of the C parameter than K indicate a slight difference in the heat of sorption of the monolayer as compared to the multilayer [60,62].

The K coefficient is related to the multilayer water. The coefficient K was lower than 1 for all the investigated sorption isotherms (Table 3), which complies with its physical impact [63]. Moreover, the obtained values of the K parameter (ranging from 0.942 to 0.900) generally indicate the high-level structured state of the multilayer water. However, the slightly lower K coefficient recorded for chitosan with higher concentrations of propolis extract could be interpreted by a less structured state of the multilayer [64].

### 3.5. Attenuated Total Reflectance Fourier Transform Infrared Spectroscopy

**Figure 4:**
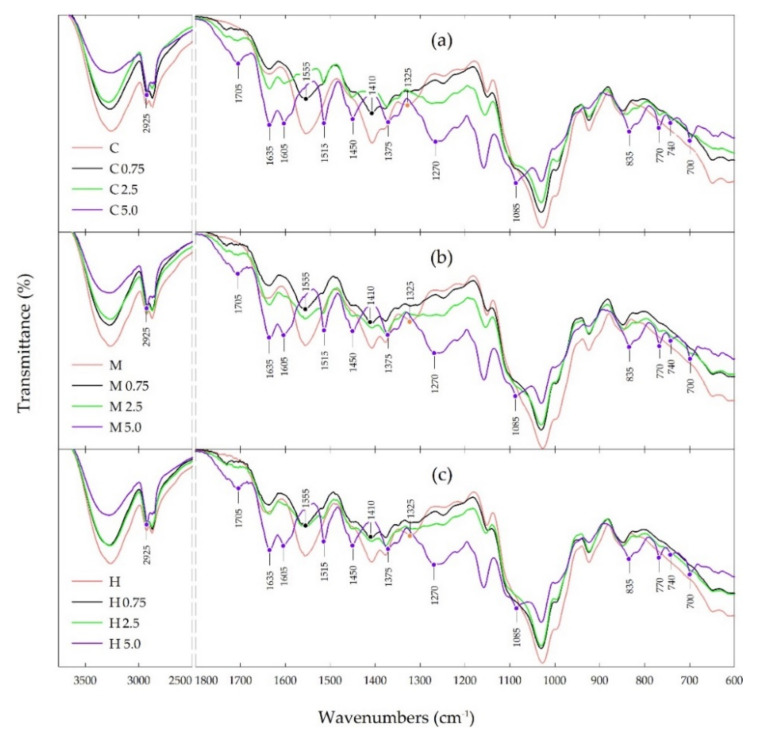
FTIR spectra of chitosan and chitosan-propolis films: (**a**) crab shells, (**b**) medium molecular weight, (**c**) high molecular weight.

The FTIR spectra of various types of chitosan films and chitosan films with propolis extract at a concentration in the range of 0.75–5.0% are presented in Figure 4.

ATR-FTIR spectroscopy was performed mainly to assess the interaction of phenolic compounds from propolis extract with the chitosan matrix in the tested films. The obtained results indicate that all types of chitosan films showed a similar pattern of the IR spectra with the same characteristic peaks, but with different intensity of transmittance. Furthermore, all the chitosan-propolis films showed the same pattern of the FTIR spectra, but the transmittance intensity of the characteristic peaks increased with an increase in the concentration of propolis used to prepare these films.

The main bands for pure chitosan films include a broad band in the range of 3500–3000 cm^−1^, related to the stretching vibration of the hydroxyl group (-OH) and amino groups (-NH), a band at 2925 cm^−1^ corresponding to CH- symmetric and asymmetric stretching vibration, a band at 1555 cm^−1^ attributed to -NH bending vibration of non-acrylated 2-aminoglucose primary amines of chitosan and a band at 1410 cm^−1^ ascribed to symmetric carboxylate anion stretching of chitosan [14,15,21,22,65,66,67].

The FTIR spectra of chitosan-propolis films showed a decrease in the intensity of the bands in the range 3500–3000 cm^−1^ and at 2925 cm^−1^, and an almost complete disappearance of the bands at 1555 and 1410 cm^−1^ compared to the pure chitosan spectra. On the other hand, in the spectra of chitosan-propolis films the appearance of new bands is observed, the intensity of which increases with increasing propolis concentration. For the chitosan-propolis films the main bands observed in the FTIR spectra include a band at 1750 cm^−1^, related to C=O stretching vibration of flavonoids, a band at 1635 cm^−1^ attributed to C=O and C=C stretching vibrations and N-H asymmetric bending vibration of aromatic rings of flavonoids and amino acids, and a bands at 1515 and 1450 cm^−1^, corresponding to stretching vibration of aromatic rings [68,69,70]. Moreover, a band at 1375 cm^−1^ coming from CH_2_- bending vibration of flavonoids, a band at 1270 cm^−1^ corresponding to C-O stretching vibration and a band at 1085 cm^−1^, related to C-C stretching vibration and C-OH bending vibration of flavonoids and secondary alcohol groups are also observed in the spectra of chitosan-propolis films [21,68,69,70]. In turn, the peaks in the range of 835–700 cm^−1^ found in the spectra of chitosan-propolis films can be ascribed to C-H aromatic rings of phenolic compounds from propolis [21].

The changes in the intensity of the bands and the appearance of new bands observed in the FTIR spectra of chitosan and chitosan-propolis films may be related to possible interactions between the chitosan matrix and propolis components, mainly phenolic compounds. Moreover, the increase in the intensity of the bands along with the increase in propolis concentration suggests greater interactions between propolis components and chitosan. The disappearance of the peak at 1655 cm^−1^ ascribed to the amino group of chitosan may be related to the interaction of the -NH group with the functional group of phenolic compounds [15]. Moreover, the narrowing of the wide band of -OH in the range of 3500–3000 cm^−1^ observed in the spectra of the pure chitosan film compared to the bands in the spectra of the chitosan-propolis films may indicate that the hydroxyl group of chitosan forms hydrogen bonds with phenolic compounds of propolis.

## 4. Conclusions

The present paper describes biological and physicochemical properties of films based on three types of chitosan (from crab shells, medium and high molecular weight chitosan) and propolis extract (in the concentration range of 0.75–5.0%). In vitro antimicrobial activity tests indicated that all pure chitosan films showed no activity against tested strains of bacteria and fungi. In turn, the addition of the propolis extract to obtain chitosan-propolis films caused an increase in their activity against Gram-positive bacteria—*B. cereus*. Moreover, the films consisted of high molecular weight chitosan and the propolis extract showed an inhibitory effect against *L. monocytogenes* and *S. aureus*. This activity could be connected with the fact that high molecular weight chitosan has a compact structure due to strong intramolecular hydrogen bonds, therefore phenolic compounds from propolis extract may be less bound to the chitosan matrix and thus may diffuse through the agar medium and inhibit bacterial growth. Moreover, both the type of chitosan and propolis concentration affected oxygen transmission rate (OTR) and water vapor transmission rate (WVTR). The best barrier properties were shown for the film consisting of high molecular weight chitosan and propolis extract added at the concentration of 5.0%. The better barrier property of high molecular weight chitosan compared to the other chitosan types could be connected with the fact that this chitosan type has a compact structure due to strong intramolecular hydrogen bonds. The results of sorption experiments showed a slight influence of the chitosan type and a significant effect of propolis extract concentration on equilibrium moisture content (EMC) of tested films. A distinct reduction of EMC value was observed for the film consisting of high molecular weight chitosan with 5.0% propolis extract. In turn, the ATR-FTIR results indicated that phenolic compounds from propolis extract interact with the chitosan matrix.

The obtained results indicate that chitosan films with an addition of propolis extract are promising materials for food packaging applications. The film consisting of high molecular weight chitosan and propolis extract (especially in the concentration of 5.0%) exhibits the greatest potential for use in food packaging thanks to its good biological and physicochemical properties, which can also be used in packaging of food containing probiotic microorganisms.

## Figures and Tables

**Table 1:** The symbols of film samples.

Type of Chitosan	Concentration of Propolis Extract (%)	Symbols
Crab shells	-	C
	0.75	C 0.75
	2.5	C 2.5
	5.0	C 5.0
Medium molecular weight	-	M
	0.75	M 0.75
	2.5	M 2.5
	5.0	M 5.0
High molecular weight	-	H
	0.75	H 0.75
	2.5	H 2.5
	5.0	H 5.0

**Table 2:** The antimicrobial activity of films based on chitosan and propolis extract.

Type of Microbe	Inhibition Zone (mm)											
	Crab Shells Chitosan (C)				Medium Molecular Weight Chitosan (M)				High Molecular Weight Chitosan (H)			
	Ethanolic Extract of Propolis (%)											
	-	0.75	2.5	5.0	-	0.75	2.5	5.0	-	0.75	2.5	5.0
**Pathogenic bacteria**												
*Lysteria* *monocytogenes*	0	0	0	0	0	0	0	0	0	17	26	26
*Staphylococcus* *aureus*	0	0	0	12	0	0	0	12	0	14	21	22
*Bacillus* *cereus*	0	12	13	13	0	12.5	13	13	0	12	13	14

Inhibition zones of all tested chitosan-based films against *E. coli*, *S.* Typhimurium, *Lb. paracasei*, *E. faecium* and *S. cerevisiae* were 0.

**Table 3:** Estimated coefficients of the GAB model (Equation (2)) for chitosan at 25 °C depending on propolis extract concentration.

Type of Chitosan	Concentration of Propolis Extract (%)	Symbol	M_m_ (kg/kg)	K	C	R^2^
Crab shells	-	C	0.176	0.922	1.150	0.99996
	0.75	C 0.75	0.129	0.931	1.112	0.99997
	2.5	C 2.5	0.095	0.931	1.324	0.99993
	5.0	C 5.0	0.069	0.900	1.143	0.99963
Medium molecular weight	-	M	0.159	0.933	1.256	0.99995
	0.75	M 0.75	0.122	0.927	1.140	0.99995
	2.5	M 2.5	0.089	0.942	1.367	0.99993
	5.0	M 5.0	0.065	0.918	1.368	0.99986
High molecular weight	-	H	0.171	0.934	1.208	0.99996
	0.75	H 0.75	0.134	0.932	1.096	0.99997
	2.5	H 2.5	0.098	0.928	1.242	0.99992
	5.0	H 5.0	0.056	0.913	1.412	0.99984

## Data Availability

The data reported in this study can be available by requesting the authors.
